# Wide-field fluorescein angiography findings in active anterior scleritis

**DOI:** 10.1186/s12348-024-00439-5

**Published:** 2024-12-18

**Authors:** Juan C. Romo-Aguas, Miguel Zavaleta-Mercado, Rashel Cheja-Kalb, Claudia Hubbe-Tena, Luz Elena Concha-del-Río

**Affiliations:** 1 Asociación para Evitar la Ceguera en México, Vicente Garcia Torres No. 46 Coyoacán, Mexico City, 04030 Mexico; 2 Inflammatory Eye Disease Clinic, Asociación para Evitar la Ceguera en México, Vicente Garcia Torres No. 46 Coyoacán, Mexico City, 04030 Mexico

**Keywords:** Anterior scleritis, Fluorescein angiography, ANCA-associated vasculitis, Wide-field angiography

## Abstract

**Objective:**

Describe the proportion of patients with wide-field fluorescein angiographic (WFFA) findings in patients with active anterior scleritis.

**Methods:**

An observational, descriptive, cross-sectional study of the WFFA findings of patients with active anterior scleritis including nodular, diffuse, or necrotizing involvement was performed. Studies were performed with the Heidelberg Spectralis module (102º). Images were saved and assessed by two masked co-authors.

**Results:**

Seventy-nine eyes from 39 patients, 31 (79%) females with a mean age of 50.5 years ± 13.98. Mean best-corrected visual acuity (BCVA) of 0.1343 ± 0.2475 logMar or 20/27. WFFA findings were observed in 39.58% of eyes with active scleritis and in 16.66% of eyes without scleritis. Systemic association was present at 50%, with ANCA-associated vasculitis being the most prevalent. WFFA findings were more frequent in cases of anterior diffuse scleritis and correlated with scleritis severity when central and peripheral leakage (*p* < 0.022) and cystoid macular edema (*p* < 0.013) were present.

**Conclusions:**

Almost 40% of eyes with anterior scleritis have WFFA findings of vascular leakage and 16% of eyes without scleritis. Retinal vascular leakage needs to be explored as a sign of local and/or systemic activity in patients with anterior scleritis and may have implications for disease severity and management.

## Introduction

 Anterior scleritis is a chronic, inflammatory scleral disease. It is a form of active vasculitis, which can appear with or without necrosis. It can be the first sign of an autoimmune disease, and 50% of cases are associated with Anti-Neutrophil Cytoplasmic Antibodies (ANCA) associated vasculitis (AAV), rheumatoid arthritis (RA), systemic lupus erythematosus (SLE), and HLA-B27 associated spondyloarthropathies [1–4]. 

Ocular vasculitic processes are often not confined to one region and can affect the retina, sclera, choroid, and/or periocular tissue. Also, it can be the first sign of an underlying systemic disease [5, 6]. 

Limited studies exist on the association between active anterior scleritis and changes in the retinal vasculature, including diseases like granulomatosis with polyangiitis (GPA), microscopic polyangiitis (MAP), RA, Behcet’s disease and Sjögren syndrome [7–11]. 

Retinal vascular involvement is secondary to the deposition of immune complexes that affect the periphery of the retina, usually with no symptoms or clinical signs. The gold standard for RV diagnosis is fluorescein angiography. In recent years, wide-field fluorescein angiography (WFFA) has gained importance for the evaluation of the periphery of the retina. Conventional angiography takes images of the central retina (30–60º), with a montage of 90º, and WFFA of 102º [7, 8]. 

WFFA in non-infectious uveitis serves as a marker of clinical inflammation, demonstrates occlusive disease, and show location patterns for location of leakage, whether central, peripheral, or diffuse [12]. 

We aimed to describe the proportion of patients with wide-field fluorescein angiographic findings in cases of active anterior scleritis.

## Methods

An observational, descriptive, cross-sectional study of the WFFA findings of patients with active anterior scleritis was conducted between March 2018 through February 2020 at the Inflammatory Eye Disease Clinic at the Asociación para Evitar la Ceguera en México. Institutional Review Board (IRB)/Ethics Committee approval was obtained, and the study was carried out in accordance with the principles of the Declaration of Helsinki.

A consecutive series of adult patients diagnosed with active anterior scleritis, whether associated with rheumatological diseases, such as RA and AAV or idiopathic were included. After assessing the inclusion criteria, relevant data on the current clinical course, best corrected visual acuity (BCVA), refractive error (low myopia: <-3.00 diopters; moderate: -3.00 to -6.00 diopters; high: >-6.00 diopters), history of any ​​rheumatological disease, time since diagnosis, treatment received, and if any systemic disease was active at the time of scleritis, were collected. Initial symptoms and signs, laterality, type of anterior scleritis (nodular, diffuse, necrotizing, and/or peripheral ulcerative keratitis) [11], and severity of scleral inflammation based on the standardized grading system (none, minimal, mild, moderate, severe, and necrotizing) were recorded [5]. To describe the affected area, a vertical line passing through meridians 6 and 12 of the cornea divided the temporal and nasal areas, and a horizontal line passing through meridians 3 and 6 divided the superior and inferior areas. Findings related to posterior pole involvement preceding or concurrent with scleritis were documented.

Laboratory results were used to confirm the systemic diagnoses (C-reactive protein, globular sedimentation rate, rheumatoid factor, anti-cyclic citrullinated peptide, ANCA, MPO, PR3, HLA-B27) and epidemiological information was retrieved from electronic medical records.

Exclusion criteria included retinal pathology (e.g., diabetic retinopathy, age-related macular degeneration), posterior synechiae that made it impossible to assess the posterior pole, chronic renal insufficiency, uncontrolled systemic arterial hypertension, and/or diabetes; pregnancy, breastfeeding, or puerperium patients; retinal vasculitis (RV) due to infectious, hematological, coagulopathy, or neoplastic etiology. We did not consider patients in whom the study could not be performed due to reasons such as allergy to fluorescein.

WFFA findings were defined as follows: central leakage (affection of macula alone), peripheral leakage (involving leakage outside the vascular arcades), central and peripheral leakage (if < 50% retinal vasculature was involved), or diffuse leakage (> 50% of the retinal vasculature involved) [12]. To describe the area affected area, a vertical line passing through the fovea divided the temporal and nasal areas, while a horizontal line passing through the macula and optic nerve divided the area into superior and inferior sections. Tortuosity, vascular occlusions, arteriovenous (AV) shunts, cystoid macular edema, optic disc hyperfluorescence, and occlusive vasculitis were also recorded.

WFFA studies were performed with the Heidelberg Spectralis instrument (Heidelberg Engineering, Heidelberg, Germany) with a 102º lens, with adequate dilation and fixation with an external fixation device. The intravenous fluorescein injection was administered into the extended arm of the patient. A joystick was used to align the illumination beam within the designated field of view, with a pre-planned photographic sequence: nine projections, starting in the posterior pole and then in the eight peripheral quadrants starting with the eye of active with anterior scleritis. Images were saved and assessed by two masked co-authors (RCK and LECR).

For statistical analysis, information was separately recorded on a spreadsheet using Microsoft Excel (Excel 2016). Data was analyzed using SPSS version 23.0 (NY: IBM Corp.). As for the statistical analysis of the descriptive variables, measures of central tendency and frequencies were carried out. Subsequently, for comparative analysis of systemic diseases, the antibody and angiographic findings were analyzed using chi-square, and a value of *p* < 0.05 was considered statistically significant. Concordance was performed with the X test and inter-rater agreement was calculated with Cohen’s kappa coefficient.

Patients or the public were not involved in the design, conduct, reporting, or dissemination plans of our research.

## Results

We included 39 patients with bilateral disease in 23%, a median age of 50.5 years ± 13.98 years, 79% females (Table 1), and a mean best-corrected visual acuity (BCVA) of 0.1343 ± 0.2475 logMAR. The mean refractive error was 0.15 ± 0.25 diopters, with mild myopia found in 29.4%, moderate myopia in 6.3%, hyperopia in 35.41%, and the rest with no refractive error.

**Table 1:** Demographic data and types of scleritis by disease

	Rheumatoid arthritis *n* (%)	AAV *n* (%)	Idiopathic *n* (%)	Total *n* (%)
Patients	7 (17.9%)	13 (33%)	19 (48.7%)	39
Gender (female/male)	7/0	11/2	13/6	31/8
Bilateral (patients)	1	3	5	9
Eyes with scleritis	8 (16.6%)	16 (33%)	24 (50%)	48
Type of scleritis				
Nodular	2 (25%)	2 (12.5%)	12 (50%)	16 (33.3%)
Diffuse	6 (75%)	9 (56.25%)	11 (45.8%)	26 (54.16%)
Necrotizing	0	5 (31.25%)	1 (4.16%)	6 (8.33%)

Five (12%) patients had associated controlled arterial systemic hypertension. A systemic autoimmune association was observed in 51% of the patients, represented by AAV in 33.3% and RA in 17.9%, with a mean duration of 16.41 ± 55.17 months. Systemic activity was found in 29.2%. Of all patients, 14.6% (*n* = 7 eyes) were treated with oral prednisone, 10.4% (*n* = 5 eyes) with methotrexate and 4.16% (*n* = 2 eyes) with rituximab.

Based on the classification of scleral inflammation, we included 48 eyes with three types of anterior scleritis: 26 eyes (54.1%) with diffuse anterior scleritis, 16 eyes (33.3%) with nodular anterior scleritis, and six eyes (12.5%) with necrotizing anterior scleritis. On fundoscopic evaluation, we found optic nerve hyperemia (*n* = 2, 4.1%), macular edema (*n* = 2, 4.1%), peripheral vascular sheathing (*n* = 8, 8.3%), and serous retinal detachment (*n* = 1, 2.05%). No patient had a history of retinal vasculitis.

The severity of scleritis severity ranged from mild to severe; there was no statistically significant difference (*p* < 0.090) when correlated with associated diseases nor in the number of quadrants affected (*p* < 0.312).

Regarding the WFFA studies, there was a moderate level of agreement between the two masked researchers, with a Cohen´s kappa of 0.664.

**Fig. 1:**
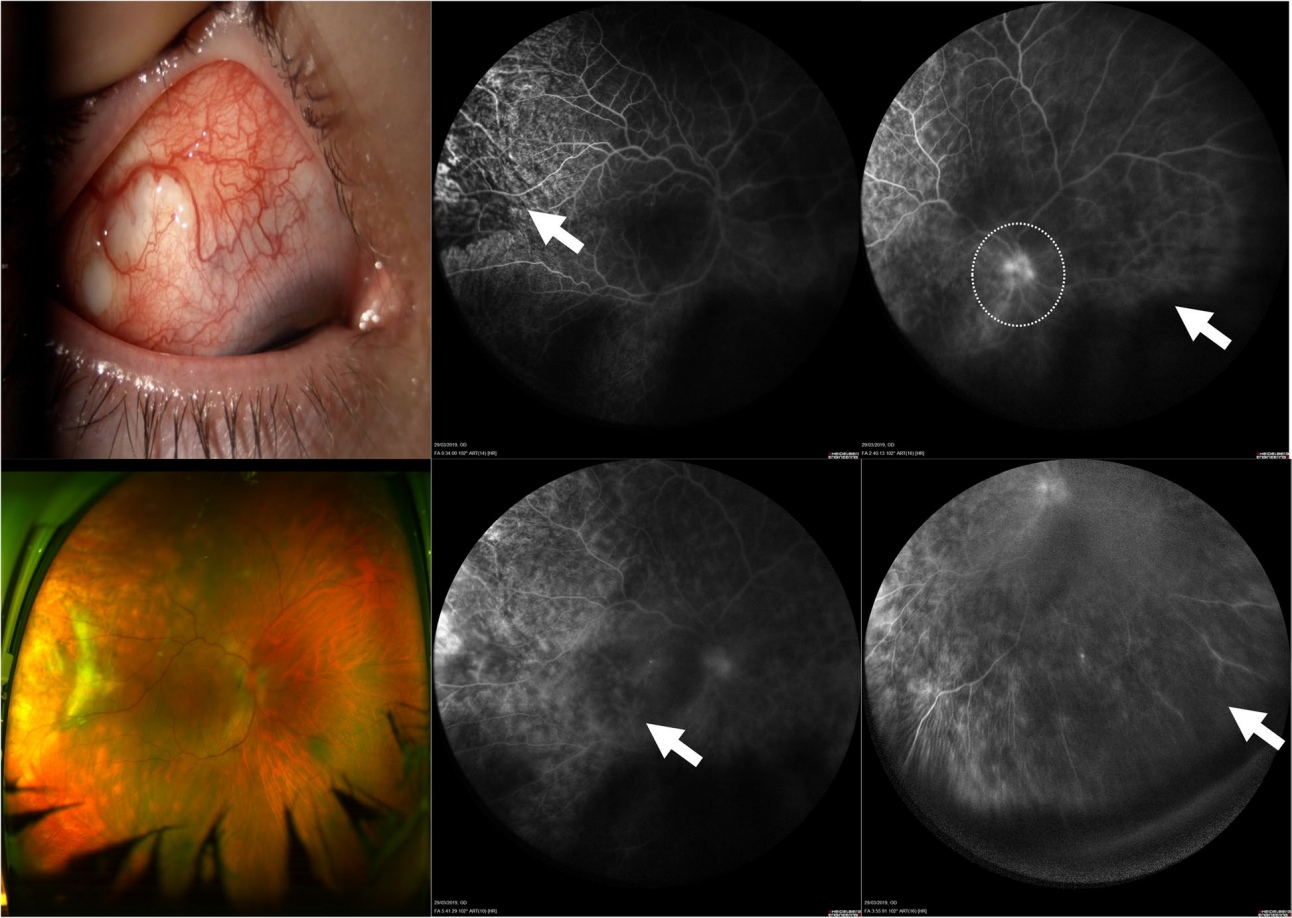
A female in her early 40s with necrotizing anterior scleritis in the right eye, associated with AAV, p-ANCA+, MPO+, with central and peripheral leakage, and cystoid macular edema (arrows) and optic nerve leakage (circle)

According to the classification, WFFA findings were observed in 14 eyes (29.16%). The most frequent finding was peripheral leakage, observed in 31.25% of cases; central and peripheral leakage was only seen in AAV (*p* < 0.001). (Table 2) (Fig. 1) WFFA findings were more frequent in anterior diffuse scleritis and correlated with scleritis severity when central and peripheral leakage was present (*p* < 0.022), as well as with cystoid macular edema (*p* < 0.013). (Tables 3 and 4)

**Table 2:** WFFA findings per eye and its correlation with systemic disease

	Rheumatoid arthritis *n* (%)	AAV *n* (%)	Idiopathic *n* (%)	Total *n* (%)
Total	8 eyes	16 eyes	24 eyes	48 eyes
Central leakage only	0	0	0	0
Central and peripheral leakage	0	1 (6.25 %)	1 (4.16%)	2 (4.16%)
Peripheral leakage only	3 (37.5%)	3 (18.75%)	9 (37.5%)	15 (31.25%)
Diffuse leakage	1 (12.5 %)	0	1 (4.16%)	2 (4.16%)
Macular edema	0	1 (6.25 %)	1 (4.16%)	2 (4.16%)
Optic disc hyperfluorescence	0	1 (6.25%)	3 (12.5%)	4 (8.33%)
Shunts	0	4 (25%)	3 (12.5%)	7 (14.58%)
Vascular occlusion	0	2 (12.5%)	0	2 (4.16%)
Vascular tortuosity	3 (37.5%)	8 (50%)	8 (33.3%)	19 (39.58%)

**Table 3:** WFFA findings and type of scleritis

	Nodular *n* = 16	Diffuse *n* = 26	Necrotizing *n* = 6	Without scleritis *n* = 30
Central leakage only	0	0	0	0
Central and peripheral leakage	0	0	2 (33.3%)	0
Peripheral leakage only	2 (12.5%)	6 (23.07%)	2 (33.3%)	5 (16.66%)
Diffuse leakage	1 (6.25%)	1 (3.84%)	0	0
Macular edema	0	1 (3.84%)	1 (16.6%)	0
Optic disc hyperfluorescence	1 (6.25%)	1 (3.84%)	2 (33.3%)	4 (13.3%)
Shunts	2 (12.5%)	2 (7.69%)	1 (16.6%)	2 (6.66%)
Vascular occlusion	0	1 (3.84%)	1 (16.6%)	0
Vascular tortuosity	3 (18.7%)	10 (38.46%)	2 (33.3%)	4 (13.3%)

**Fig. 2:**
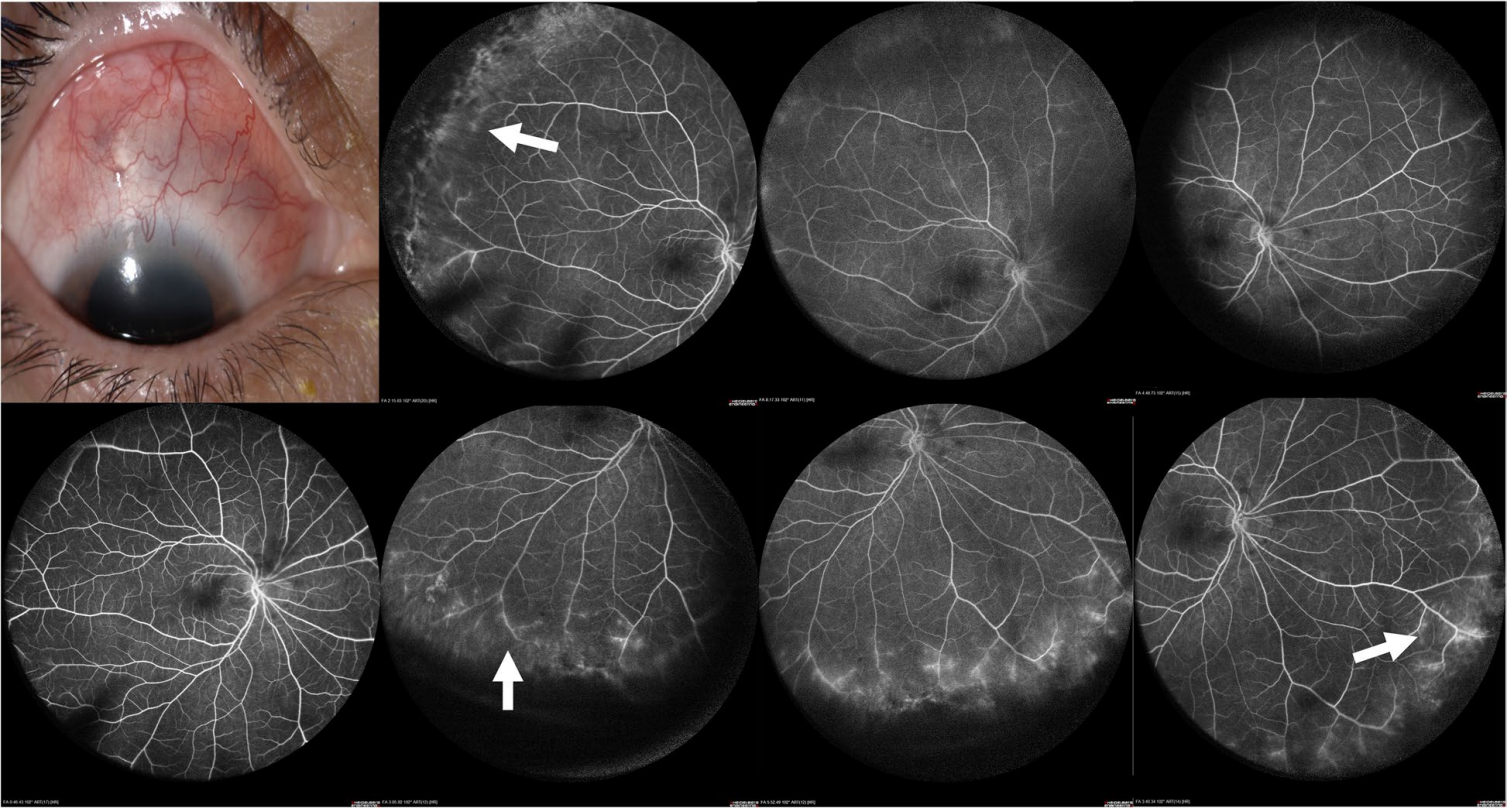
A female in her late 40s with a diagnosis of RA and anterior nodular scleritis. WFFA in the late arteriovenous phase with the presence of peripheral vascular leakage and parietal staining in upper temporal, lower temporal, and lower nasal quadrants (arrow)

There were no statistically significant differences in demographic or clinical variables, as in the positivity of acute phase reactants like CPR (*p* = 0.569) and ESR (*p* = 1). There was a correlation between the clinical variable of the number of quadrants with scleritis and with WFFA findings (*p* < 0.027). No correlation was found between the location of scleritis and the retinal affected area in WFFA (*p* = 0.472), as seen in Fig. 2, nor between peripheral vascular occlusion and necrotizing anterior scleritis (*p* = 0.237) or myopia (*p* = 0.125).

Other angiographic findings included peripheral occlusion in AAV (4.16%), shunts in seven eyes (14.58%), CME in two eyes (4.16%), optic disc hyperfluorescence in four eyes (8.33%), and tortuosity in 19 eyes (24.3%); no eye had occlusive vasculitis. (Tables 2 and 3).

**Table 4:** WFFA findings and scleritis severity

	Minimal *n*=1	Mild *n*=8	Moderate *n*=26	Severe *n*=11	Necrotizing *n*=2	Total *n*=48	*p*
Central leakage only	0	0	0	0	0	0	-
Central and peripheral leakage	0	0	1	0	1	2	.022
Peripheral leakage only	0	4	3	3	0	10	.161
Diffuse leakage	0	0	1	1	0	2	.886
Macular edema	0	0	0	1	1	2	.013
Optic disc hyperfluorescence	0	0	1	1	2	4	.004
Shunts	0	1	3	1	0	5	.979
Vascular occlusion	0	0	1	1	0	2	.886
Vascular tortuosity	1	1	8	4	1	15	.410

**Fig. 3:**
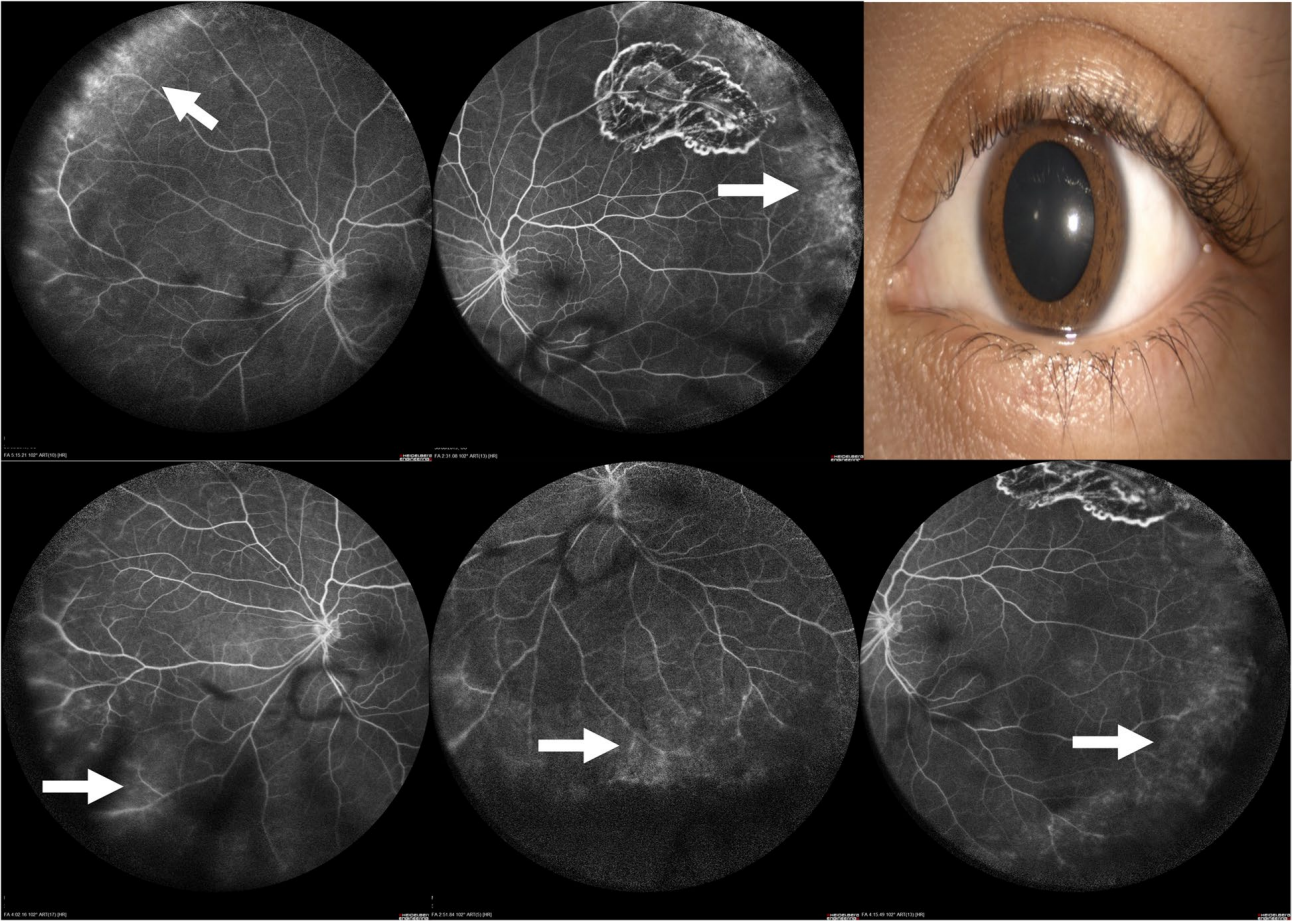
Contralateral eye without scleritis of the patient in Fig. 2. WFFA in the late arteriovenous phase with the presence of peripheral vascular leakage and parietal staining in lower and upper nasal quadrants (arrow)

During the evaluation of contralateral eyes without scleritis, we observed that 16.6% presented at least one angiographic sign, with peripheral vascular leakage being the most frequent finding. (Table 3) (Fig. 3) No statistical difference was found in eyes with and without scleritis and WFFA findings (*p* < 0.210).

## Discussion

This is the first study that systematically evaluates the potential of WFFA in diagnosing and treating active anterior scleritis, where one-third of the patients presented with retinal findings. Studying the peripheral retinal area has helped to detect novel peripheral pathology in numerous diseases.

Now we have new diagnostic tools like WFFA to visualize the retinal and choroidal vasculature. The findings can reflect systemic inflammatory processes that affect these vascular structures. WFFA can identify areas of vascular leakage, perivascular sheathing, and occlusions, which are suggestive of active vasculitis, providing important diagnostic and monitoring information. Still, we should keep in mind that there is not always a clinical-angiographic correlation. Another advantage is that vascular changes are frequently found in the retina´s periphery, and standard field cameras (30–60º) often cannot capture them.

Retinal angiography is not frequently used as a routine diagnostic test for scleritis. In certain cases, it can help diagnose associated complications or rule out other conditions that may contribute to the inflammation [7, 8, 10]. 

Therefore, we conducted a study to determine whether WFFA allows us to find changes in patients with active anterior scleritis. It is important to note that retinal angiography is an invasive procedure that carries some risks and is used when other less invasive diagnostic tests have failed to provide a clear diagnosis or when there is a specific concern that can be addressed.

In normal eyes, WFFA can assess vascular perfusion of the retina, leakage, or abnormal vessel morphology. Vascular leakage in the far peripheral retina is reported in 19.8%, [13, 14] and it may show abnormal blood vessel permeability. Vascular leakage is a common finding in eyes with uveitis; peripheral vascular leakage prevalence may vary depending on age, type of uveitis, and severity. It ranges from 27% in many uveitis [12] to 75.7% in Behcet´s disease [13], with intermediate values reported for conditions such as intermediate uveitis (29%), [15] anterior uveitis (42%), [16] juvenile idiopathic arthritis (64.86%), [17] and tubulointerstitial nephritis and uveitis (TINU) (72.2%) [18]. Additionally, it may be more likely to occur in eyes with active inflammation (55% vs. 31%) [16]. In our study, we observed it in a smaller proportion (19.2%), probably because our patient’s primary site of inflammation was the sclera and presented mild active intraocular inflammation.

A retinal examination is required in all patients with scleritis, as RV at onset is typically asymptomatic until vision loss occurs, with no unusual fundus findings. RV is an extra-articular manifestation of rheumatic diseases and can be the first finding of systemic activity in a patient with non-specific rheumatologic presentations [4]. Rheumatologists have attempted to incorporate information on end-organ involvement, to develop a universal scoring system for diagnosing, staging, and treating these conditions [4]. 

RV can be present in all autoimmune diseases, mainly in RA, SLE, and AAV, and it is associated with increased mortality [1, 4]. RV involves endothelial inflammation, leading to vascular occlusion and eventual ischemia.

The frequency of RV findings associated with rheumatologic diseases has been reported to be up to 12% with conventional angiography [19] and 67.7% with ultra-wide-field angiography [20]. The difference between these last two studies and ours is that their association refers to RV as an isolated finding, and not in combination with active anterior scleritis. RV is usually related to an inflammatory breakdown of the retinal blood-brain barrier. Eyes with RV manifest focal, segmental, or diffuse vascular leakage [12]. It is important to determine whether there is endothelial dysfunction or if active inflammation is secondary to retinal blood vessel disruption, with focal leakage from the retinal vasculature, or obstruction of the vascular lumen [4]. Peripheral vascular leakage correlates with patients requiring additional immunosuppression, which may influence management decisions versus standard-of-care treatment [4]. 

We found retinal leakage in WFFA, along with CME and vascular occlusion, indicating complications of chronic inflammation. These complications are more frequent in AAV and may not been detected without imaging [8, 20, 21]. Few studies have analyzed retinal and choroidal vessels in patients with scleritis. It is postulated that necrotizing scleritis represents a primary vasculitis affecting the deep episcleral vessels, while non-necrotizing scleritis may involve a delayed hypersensitivity reaction [22]. In a microscopical examination of eyes with necrotizing scleritis, evidence shows endarteritis and periarteritis of the thickened choroidal vessels, especially associated with the scleral reaction [23]. 

Further, we found 24.3% of venous tortuosity in the WFFA. Venous tortuosity is observed in all types of scleritis and related systemic diseases. Many retinal pathologies consider tortuosity a negative risk factor, and modifications in retinal vessels give clues about disease severity and changes in disease activity [24]. For example, in diabetes, increased vessel tortuosity relates to hemodynamic alterations caused by the disease, including disturbed blood flow, tissue hypoxia, endothelial dysfunction, and increased levels of VEGF [25, 26]. Studies have reported associations between endothelial dysfunction and conditions such as antiphospholipid syndrome, human immunodeficiency virus, and RA. This endothelial phenomenon can occur at macrovascular and microvascular levels and has a prognostic value for cardiovascular events. For example, in RA, endothelial function improved in patients whose disease activity responded to treatment [25, 27, 28]. Understanding the associations between venous tortuosity with systemic diseases is essential in the treatment and prognosis of scleritis and related conditions.

Scleritis must be properly diagnosed to guarantee accurate and sufficient treatment to improve outcomes. The sclera is more vulnerable to inflammation because of vascular circulations [11]. Active scleritis in patients with autoimmune diseases can be a manifestation of systemic activity of the underlying autoimmune condition. Flares or worsening of scleritis often parallels increased activity or exacerbations of the systemic autoimmune disease [3]. Our study detected a systemic association with active anterior scleritis in 50% of the cases, consistent with previous literature findings [29]. The most commonly associated disease was AAV, a primary systemic vasculitis involving small vessels and it was the only one with peripheral vascular occlusion on WFFA in our study. Disease activity, including extension and subsequent damage from ischemia and necrosis, should be considered when establishing if organ-threatening vasculitis exists in addition to systemic disease [3, 30]. The second commonly associated disease was RA. In RA, elevated levels of inflammatory and endothelial dysfunction markers contribute to subclinical vasculitis due to systemic activity. This has also been seen in the choroid, with an increase in choroidal thickness [31]. A previous study described RV in 18% of patients lacking clinical signs of RV [32]. Also, in rheumatoid subclinical vasculitis, there can be a decrease in blood supply, along with a thinned choroid layer, which can produce ischemia in the foveal avascular zone [33]. 

WFFA has the potential to provide insights into the underlying systemic disease processes associated with scleritis and can help with treatment-related decision-making. The correlation between active anterior scleritis and changes in WFFA emphasizes the connection between ocular inflammation and systemic diseases. Therefore, patients with anterior scleritis must be accurately evaluated for systemic diseases. In addition, detecting changes in retinal angiography could quantify the response to treatment and the disease course over time [4, 12]. 

Our study has multiple strengths. We showed that posterior pole involvement occurs simultaneously in eyes with and without active anterior scleritis. This can play an important role in deciding whether to initiate or escalate treatment and can affect short-term or long-term morbidity and mortality. However, the study also has some limitations. The first is the small sample size. Second, there may be a possibility for selection bias, given that our hospital is a tertiary referral center. Third, this being as a cross-sectional study, we do not have the evolution of retinal findings or whether they improved with the established treatment. Finally, we did not have a control group with to compare the WFFA findings with.

## Conclusions

WFFA is a valuable study to detect changes in patients with active anterior scleritis, as well as in 16.66% of eyes without scleritis. This study demonstrates the benefits of evaluating retinal vascular perfusion, leakage, and vessel morphology in anterior scleritis. The presence of vascular leakage reported in the peripheral retina highlights the potential of WFFA in identifying abnormal blood vessel permeability, which may influence disease severity and treatment, thus improving clinical outcomes and providing insights into the ocular and systemic manifestations of the disease. Further studies are needed to confirm these findings and explore the full benefits of WFFA utility in this population.

## Data Availability

No datasets were generated or analysed during the current study.

## References

[1] Kubal AA, Perez VL (2010) Ocular manifestations of ANCA-associated Vasculitis. Rheum Dis Clin North Am 36:573–586 20688251 10.1016/j.rdc.2010.05.005

[2] Dammacco R (2018) Systemic lupus erythematosus and ocular involvement: an overview. Clin Exp Med 18:135–149 29243035 10.1007/s10238-017-0479-9

[3] Foster CS (2013) Ocular manifestations of the potentially lethal rheumatologic and vasculitic disorders. J Fr Ophtalmol 36:526–532 23688612 10.1016/j.jfo.2012.12.004

[4] Androudi S et al (2013) Retinal vasculitis in rheumatic diseases: an unseen burden. Clin Rheumatol 32:7–13 22955636 10.1007/s10067-012-2078-1

[5] Sen HN, Sangave AA, Goldstein DA, Suhler EB, Cunningham D et al (2011) A standardized grading system for scleritis. Ophthalmology 118(4):768–771 21093921 10.1016/j.ophtha.2010.08.027 PMC3070789

[6] Fong LP, de la Maza MS, Rice BA, Kupferman AE, Foster CS (1991) Immunopathol scleritis. Ophthalmol 98:472–479 10.1016/s0161-6420(91)32280-2 1828871

[7] Chi Y, Guo C, Peng Y, Qiao L, Yang L (2015) A prospective, observational study on the application of ultra-wide-field angiography in the evaluation and management of patients with anterior uveitis. PLoS One 10:e0122749 25815841 10.1371/journal.pone.0122749 PMC4376869

[8] Agarwal A, Afridi R, Agrawal R, Do DV, Gupta V, Nguyen QD (2017) Multimodal imaging in retinal vasculitis. Ocul Immunol Inflamm 25:424–433 28696172 10.1080/09273948.2017.1319494

[9] Sandhu RK, Adams T, Sibley C, Suhler EB, Kim DH (2016) Granulomatosis with Polyangiitis (GPA) presenting with Frosted Branch Angiitis. Retin Cases Br Rep 10:249–251 10.1097/ICB.0000000000000242 26579594

[10] Diaz-Valle D, Gomez-Gomez A, Pascual-Martin A (2016) Bilateral scleritis and retinal vasculitis in microscopic polyangiitis. Ophthalmology 123:2553 27871395 10.1016/j.ophtha.2016.06.050

[11] Watson P, Hayreh S (1976) Scleritis and episcleritis. Br J Ophthalmol 60:163–191 1268179 10.1136/bjo.60.3.163 PMC1042706

[12] Pecen P, Petro KF, Baynes K, Ehlers JP, Lowder CY, Srivastava MD (2017) Peripheral findings and retinal vascular leakage on Ultra-widefield Fluorescein Angiography in patients with Uveitis. Ophthalmol Retin 1:428–434 10.1016/j.oret.2017.01.016 31047575

[13] Mesquida M, Llorenç V, Fontenla JR, Navarro MJ, Adán A (2014) Use of ultra-wide-field retinal imaging in the management of active Behcet retinal vasculitis. Retina 34:2121–2127 24946103 10.1097/IAE.0000000000000197

[14] Lu J, Mai G, Luo Y, Li M, Cao D, Wang X, Yan H, Sadda SR, Lu L (2017) Appearance of far peripheral retina in normal eyes by ultra-widefield fluorescein angiography. Am J Ophthalmol 173:84–90 27693444 10.1016/j.ajo.2016.09.024

[15] Laovirojjanakul W, Acharya N, Gonzales JA (2019) Ultra-widefield Fluorescein angiography in intermediate uveitis. Ocul Immunol Inflamm 27(3):356–361 29040047 10.1080/09273948.2017.1371764

[16] Chi Y, Guo C, Peng Y, Qiao L, Yang L (2015) A prospective, observational study on the application of ultra-wide-field angiography in the evaluation and management of patients with anterior uveitis. PLoS ONE 27(3):e0122749 10.1371/journal.pone.0122749 PMC4376869 25815841

[17] Tripathy K, Ying H, Maldonado AP, Filipowicz A, Kaya M, Seymen Z, Anesi SD, Chang PE, Foster CS (2022) Widefield fundus fluorescein angiography features of uveitis associated with juvenile idiopathic arthritis. Ocul Immunol Inflamm 19:829–838 10.1080/09273948.2020.1834586 33264037

[18] Cao JL, Srivastava SK, Venkat A, Lowder CY, Sharma S (2020) Ultrawidefield fluorescein angiography and optical coherence tomography findings in tubulointerstitial nephritis and uveitis syndrome, ophthalmology retina. Ophthalmol Retina 4(2):189–197 31708486 10.1016/j.oret.2019.08.012

[19] Abu El-Asrar AM, Herbort CP, Tabbara KF (2005) Retinal vasculitis. Ocul Immunol Inflamm 13:415–433 16321886 10.1080/09273940591003828

[20] Leder HA, Campbell JP, Sepah YJ, Gan T, Dunn JP, Hatef E et al (2013) Ultra-wide-field retinal imaging in the management of non-infectious retinal vasculitis. J Ophthalmic Inflamm Infect 3:1–6 23514542 10.1186/1869-5760-3-30 PMC3610112

[21] Campbell JP, Beardsley RM, Palejwala NV, Baynham JT, Lin W et al (2015) Peripheral vascular leakage in uveitis: clinical and angiographic findings. Ophthalmology 122(6):1269–1270 25769846 10.1016/j.ophtha.2015.01.011

[22] Bernauer W, Watson P, Daicker B, Lightman S (1994) Cells perpetuating the inflammatory response in scleritis. Br J Opthalmol 78:381–385 10.1136/bjo.78.5.381 PMC504792 8025072

[23] Moore JG, Sevel D (1966) Corneo-scleral ulceration in periarteritis nodosa. Br J Ophthal 50:651–655 4380860 10.1136/bjo.50.11.651 PMC506292

[24] Hart W, Goldbaum M, Cote BK, Nelson M (1999) Measurement and classification of retinal vascular tortuosity. Int J Med Inf 53:239–252 10.1016/s1386-5056(98)00163-4 10193892

[25] Kucukkomurcu E, Unal AU, Esen F, Ozen G, Direskeneli H, Kazokoglu H (2020) Ocular posterior segment involvement in patients with antiphospholipid syndrome and systemic Lupus Erythematosus. Ocul Immunol Inflamm 28:86–91 30556792 10.1080/09273948.2018.1552759

[26] Sasongko M, Wong TY, Nguyen TT, Cheung VY, Shaw JE, Wang JJ (2011) Retinal vascular tortuosity in persons with diabetes and diabetic retinopathy. Diabetologia 54:2409–2416 21625945 10.1007/s00125-011-2200-y

[27] Agarwal A, Invernizzi A, Acquistapace A, Riva A, Agrawal R, Jain S, Aggarwal K, Gupta V, Dogra D, Singh R (2017) Analysis of Retinochoroidal vasculature in human immunodeficiency virus infection using spectral-domain OCT angiography. Ophthalmol Retin 1:545–554 10.1016/j.oret.2017.03.007 31047450

[28] Foster W, Lip G, Raza K, Carruthers D, Blann A (2012) An observational study of endothelial function in early arthritis. Eur J Clin Invest 42:510–516 21985471 10.1111/j.1365-2362.2011.02607.x

[29] Barbosa-Cobos RE, Recillas-Gispert C, Arellanes-García L (2011) Manifestaciones oculares de las vasculitis primarias sistémicas. Reumatol Clin 7:12–17 10.1016/j.reuma.2011.10.003 22119276

[30] Datoo O’Keefe GA, Rao N (2021) Retinal vasculitis: a Framework and proposal for a classification system. Surv Ophthalmol 66(1):54–67 32450158 10.1016/j.survophthal.2020.05.004

[31] Tetikoglu M, Temizturk F, Sagdik HM, Aktas S, Ozcura F et al (2017) Evaluation of the Choroid, Fovea, and retinal nerve Fiber layer in patients with rheumatoid arthritis. Ocul Immunol Inflamm 25(2):210–214 26717269 10.3109/09273948.2015.1095303

[32] Giordano N, D’Ettorre M, Biasi G, Fioravanti A, Moretti L, Marcolongo R (1990) Retinal vasculitis in rheumatoid arthritis: an angiographic study. Clin Exp Rheumatol 8(2):121–125 2338009

[33] Ayar K, Can ME, Koca N, Celij DS (2021) Evaluation of retinal vascularization by optical coherence tomography angiography (OCTA) in rheumatoid arthritis, and its relationship with disease activity. Mod Rheumatol 31(4):817–826 32997565 10.1080/14397595.2020.1830740

